# Bovine Kobuviruses from Cattle with Diarrhea

**DOI:** 10.3201/eid1406.070784

**Published:** 2008-06

**Authors:** Pattara Khamrin, Niwat Maneekarn, Supatra Peerakome, Shoko Okitsu, Masashi Mizuguchi, Hiroshi Ushijima

**Affiliations:** * University of Tokyo, Tokyo, Japan; † Aino Health Science Center, Tokyo; ‡ Chiang Mai University, Chiang Mai, Thailand

**Keywords:** Bovine kobuvirus, diarrheic cattle, Chiang Mai, Thailand, letter

**To the Editor:** A new species of kobuvirus, named U-1 strain, was first recognized in 2003 as a cytopathic contaminant in a culture medium of HeLa cells that had been used for >30 years in the laboratory (*1*). The RNA genome of the U-1 strain comprises 8,374 nucleotides; the genome organization is analogous to that of picornaviruses. Morphologically, the U-1 strain resembles the Aichi virus, but genetically it is distinct (*1*). Therefore, the U-1 strain is classified as a new species of genus *Kobuvirus* in the family *Picornaviridae*, and it is called *bovine kobuvirus* (*1*). To date, the genus *Kobuvirus* consists of 2 species, *Aichi virus* and *bovine kobuvirus* (*2*). The Aichi virus is associated with acute gastroenteritis in humans (*3*–*5*); bovine kobuvirus infection has been detected only in cattle (*1*).

Only 1 report has described the discovery and epidemiologic features of bovine kobuvirus (*1*). Of serum samples from 72 healthy cattle, 43 (59.7%) were positive for neutralizing antibody against bovine kobuvirus U-1 standard strain at a titer of >16. In addition, 12 (16.7%) of 72 stool samples collected from the cattle were positive for the bovine kobuvirus genome by reverse transcription–PCR (RT-PCR) (*1*). This finding suggested that bovine kobuvirus is common and that the virus particles could be detected in the stool samples of infected cattle. We therefore conducted an epidemiologic survey of bovine kobuvirus and report detection of this virus in stool samples from calves with diarrhea during 2001–2004 in Chiang Mai Province, Thailand.

From November 2001 to July 2004, a total of 72 fecal specimens were collected. The age of the calves ranged from 7 to 49 days. The presence of bovine kobuvirus in fecal specimens was detected by using RT-PCR with a protocol modified from the method described by Yamashita et al. (*1*). All the bovine kobuvirus strains detected in our study were analyzed further by direct sequencing of their PCR amplicons with the BigDye Terminator Cycle Sequencing Kit (Applied Biosystems, Foster City, CA, USA) on an automated sequencer (ABI 3100, Applied Biosystems). The nucleotide sequences of these portions were compared with those of reference strains available in the GenBank database by using BLAST (*6*). Phylogenetic and molecular evolutionary analyses were conducted by using MEGA version 3.1 (*7*). The nucleotide sequences of bovine kobuvirus strains described in this study were deposited in GenBank under accession nos. EF659450–EF659455.

The bovine kobuvirus was detected by the RT-PCR screening method in 6 (8.3%) of the 72 fecal specimens collected. The partial 3D regions of all 6 bovine kobuviruses exhibited highly conserved sequences of 99.3%–100% nucleotide and 100% amino acid identities to each other. In addition, by searching for the closest sequences in the databank, we found that these sequences were closely related to all 13 bovine kobuvirus reference strains available in the GenBank database (U-1, K-35, K-36, K-49, K-38, N-5, K-3, K-4, K-6, K-60, N-2, K-44, and K-55); nucleotide and amino acid sequence identities ranged from 91.0% to 95.0% and 97.4% to 99.4%, respectively. Phylogenetic analysis of partial 3D nucleotide sequences of our bovine kobuvirus strains, together with those of all published bovine kobuvirus reference strains available in the GenBank database, is shown in the Figure. The Aichi virus standard strain was included in the tree as an outlier virus. The phylogenetic tree confirmed that all 6 virus strains belonged to bovine kobuvirus and formed a tight cluster in a monophyletic branch with other published bovine kobuvirus reference strains, but they were distantly related to the Aichi virus standard strain.

**Figure:**
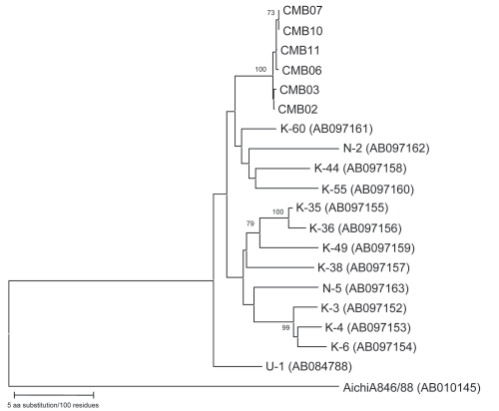
Phylogenetic analysis of the partial nucleotide sequence encoding the 3D region of bovine kobuviruses isolated in this study and other reference strains recognized to date. The tree was generated on the basis of the neighbor-joining method using the MEGA 3.1 program (*7*). Scale bar indicates branch length for a 5% nucleotide difference.

Detection and characterization of bovine kobuvirus strains from different geographic areas are important for understanding the worldwide distribution, heterogeneity, and association of bovine kobuvirus with enteric disease in cattle. Our findings indicate the role of kobuviruses in diarrheal disease in cattle and provide additional information on their relationship to bovine kobuviruses reported previously.
